# Supplementary material for: Prevalence and associated factors of non-communicable chronic diseases among university academics in Jordan
Source: PLoS One. 2024 Aug 13;19(8):e0304829. doi: 10.1371/journal.pone.0304829 (PMC11321547; doi:10.1371/journal.pone.0304829)
Supplement: S1 File — (PDF) [file pone.0304829.s001.pdf]

Stanford Patient Education Research Center
Stanford University School of Medicine

SAMPLE QUESTIONNAIRE

CHRONIC DISEASE

August 2007

You may use all or parts of the questionnaire at no charge without permission

Stanford Patient Education Research Center
1000 Welch Road, Suite 204
Palo Alto CA 94304
(650) 723-7935 voice • (650) 725-9422 fax
http://patienteducation.stanford.edu
self-management@stanford.edu

Name: ________________________________ Today's date: ____________

Address: ____

City, state, zip: ____

Telephone: home (____) ________ - ______________ Date of birth: ____________________

work (____) ________ - ______________ Sex *(circle)*: Female Male

Background

1. Ethnic origin *(check **only one**)*:

- ❑ White not Hispanic
- ❑ Black not Hispanic
- ❑ Hispanic
- ❑ Asian or Pacific Islander
- ❑ Filipino
- ❑ American Indian/Alaskan Native
- ❑ Other: ____________________

2. Please circle the ***highest*** year of school completed:

1 2 3 4 5 6	7 8 9 10 11 12	13 14 15 16	17 18 19 20 21 22	23+
(primary)	*(high school)*	*(college/university)*	*(graduate school)*	

3. Are you currently *(check **only one**)*:

- ❑ Married
- ❑ Single
- ❑ Separated
- ❑ Divorced
- ❑ Widowed

4. Please indicate below which chronic condition(s) you have:

- ❑ Diabetes
- ❑ Asthma
- ❑ Emphysema or COPD
- ❑ Other lung disease *Type of lung disease:*____________________
- ❑ Heart disease *Type of heart disease:*____________________
- ❑ Arthritis or other rheumatic disease *Specify type:*____________________
- ❑ Cancer *Type of cancer:*____________________
- ❑ Other chronic condition *Specify:*____________________

General Health

1. In general, would you say your health is:

(Circle one)

Excellent 1

Very good.............................2

Good.....................................3

Fair 4

Poor 5

Symptoms

How much time during the **past 2 weeks**...

	**None of the time**	**A little of the time**	**Some of the time**	**A good bit of the time**	**Most of the time**	**All of the time**
1. Were you discouraged by your health problems?	0	1	2	3	4	5
2. Were you fearful about your future health?	0	1	2	3	4	5
3. Was your health a worry in your life?	0	1	2	3	4	5
4. Were you frustrated by your health problems?	0	1	2	3	4	5

1. We are interested in learning whether or not you are affected by fatigue. Please *circle* the *number* below that describes your **fatigue** in the **past 2 weeks:**

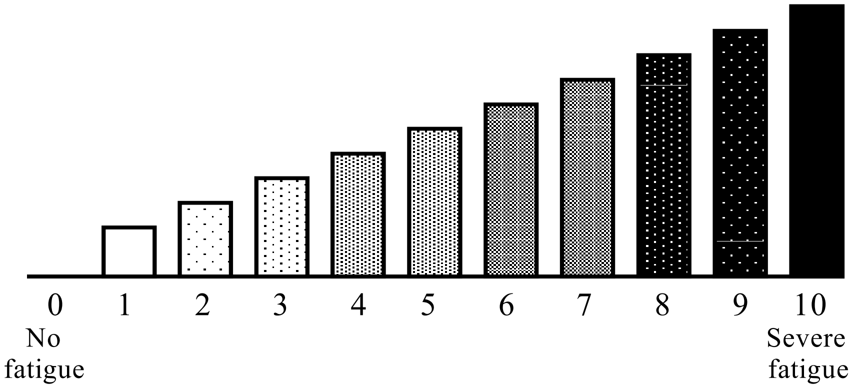

2. We are interested in learning whether or not you are affected by shortness of breath. Please *circle* the *number* below that describes your **shortness of breath** in the **past 2 weeks:**

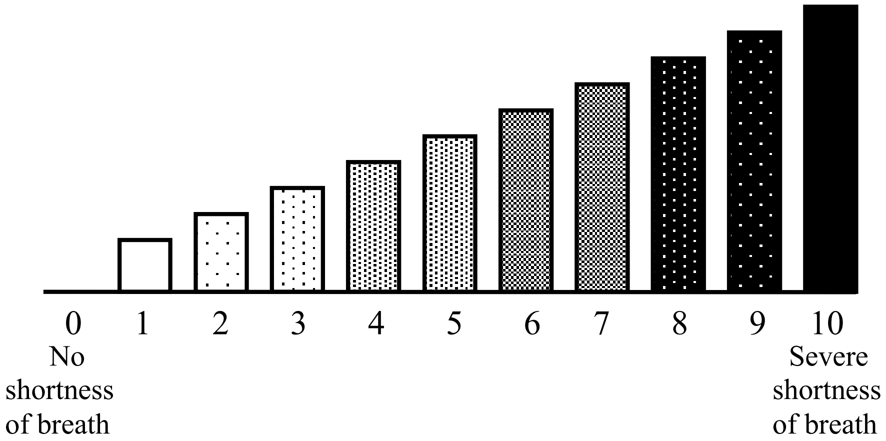

3. We are interested in learning whether or not you are affected by pain. Please *circle* the *number* below that describes your **pain** in the **past 2 weeks.**

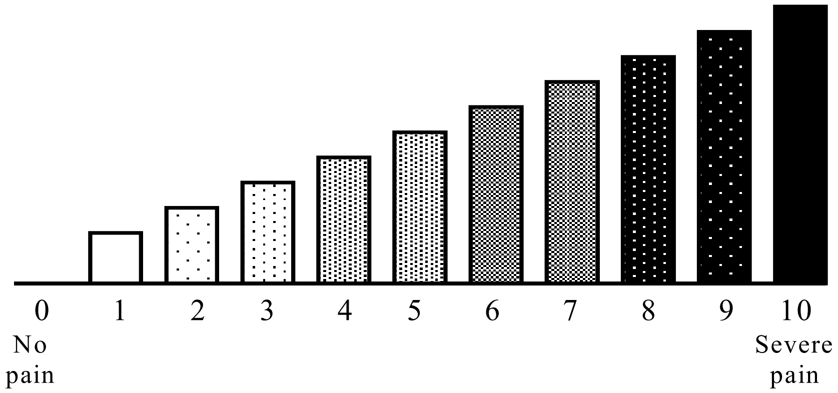

Physical Activities

During the past week, even if it was not a typical week for you, how much **total** time *(for the **entire week**)* did you spend on each of the following? *(Please circle **one** number for each question.)*

	none	less than 30 min/wk	30-60 min/wk	1-3 hrs per week	more than 3 hrs/wk
1. Stretching or strengthening exercises (range of motion, using weights, etc.)	0	1	2	3	4
2. Walk for exercise	0	1	2	3	4
3. Swimming or aquatic exercise	0	1	2	3	4
4. Bicycling (including stationary exercise bikes)	0	1	2	3	4
5. Other aerobic exercise equipment (Stairmaster, rowing, skiing machine, etc.)	0	1	2	3	4
6. Other aerobic exercise *Specify*________________________	0	1	2	3	4

Confidence About Doing Things

For each of the following questions, please ***circle*** the number that corresponds with your **confidence** that you can do the tasks regularly at the present time.

How confident are you that you can...

1. Keep the fatigue caused by your disease from interfering with the things you want to do?	not at all confident	1	2	3	4	5	6	7	8	9	10	totally confident
2. Keep the physical discomfort or pain of your disease from interfering with the things you want to do?	not at all confident	1	2	3	4	5	6	7	8	9	10	totally confident
3. Keep the emotional distress caused by your disease from interfering with the things you want to do?	not at all confident	1	2	3	4	5	6	7	8	9	10	totally confident
4. Keep any other symptoms or health problems you have from interfering with the things you want to do?	not at all confident	1	2	3	4	5	6	7	8	9	10	totally confident

How confident are you that you can...

5. Do the different tasks and activities needed to manage your health condition so as to reduce your need to see a doctor?	not at all confident	1	2	3	4	5	6	7	8	9	10	totally confident
6. Do things other than just taking medication to reduce how much your illness affects your everyday life?	not at all confident	1	2	3	4	5	6	7	8	9	10	totally confident

Daily Activities

During the **past 2 weeks**, how much... *(Circle **one**)*

	**Not at all**	**Slightly**	**Moderately**	**Quite a bit**	**Almost totally**
1. Has your health interfered with your normal social activities with family, friends, neighbors or groups?	0	1	2	3	4
2. Has your health interfered with your hobbies or recreational activities?	0	1	2	3	4
3. Has your health interfered with your household chores?	0	1	2	3	4
4. Has your health interfered with your errands and shopping?	0	1	2	3	4

Only one more page to go!

Medical Care

1. When you **visit your doctor**, how often do you do the following *(please circle **one** number for each question):*

	Never	Almost never	Some-times	Fairly often	Very often	Always
a. Prepare a list of questions for your doctor	0	1	2	3	4	5
b. Ask questions about the things you want to know and things you don't understand about your treatment............	0	1	2	3	4	5
c. Discuss any personal problems that may be related to your illness	0	1	2	3	4	5

2. **In the past 6 months**, how many times did you visit a physician?
*Do **not** include visits while in the hospital or the hospital emergency department…*__________ visits

3. **In the past 6 months**, how many times did you go to a **hospital** emergency department?..__________ times

4. **In the past 6 months**, how many TIMES were you hospitalized for one night or longer? ....__________ times

   a. How many total NIGHTS did you spend in the hospital **in the past 6 months**?..__________ nights

   b. Were any of these hospitalizations at a skilled nursing facility, convalescent hospital, or other minimum care facility? *(circle)* .... Yes No

Thank you for your help!
