# Supplementary material for: Prevalence and associated factors of non-communicable chronic diseases among university academics in Jordan
Source: PLoS One. 2024 Aug 13;19(8):e0304829. doi: 10.1371/journal.pone.0304829 (PMC11321547; doi:10.1371/journal.pone.0304829)
Supplement: S2 File — (DOC) [file pone.0304829.s002.doc]

**استبانة حول تقييم الامراض المزمنة لموظفي الجامعة الأكاديميين في الاردن**

**Prevalence and Associated Factors of Non-communicable chronic diseases Among University Academics in Jordan**

**البقاء بصحة جيدة يعد امرا صعبا عندما تعاني من مرض مزمن. نود ان نقيم وضعك الصحي كشخص يعاني من مرض مزمن او أكثر وإذا لا يوجد لديك أي مرض مزمن سنقيم وضعك الصحي بشكل عام. اجابتك سوف تعامل بسرية تامة ولن نشاركها مع أي شخص**

**أولا: المعلومات الشخصية**

1. **الجنس**:
2. . ذكر
3. . انثى
4. **العمر**:
5. 20-30 سنة
6. 31-40 سنة
7. 41-60 سنة
8. أكثر من 60 سنة
9. **الوزن: الطول:**
10. **الرتبة العلمية**:
11. أستاذ دكتور
12. أستاذ مشارك
13. أستاذ مساعد
14. مدرس
15. مساعد بحث وتدريس
16. أخرى اذكرها
17. **التحصيل العلمي**:
18. بكالوريوس
19. ماجستير
20. دكتوراه
21. **الحالة الاجتماعية:**
22. متزوج
23. غير متزوج
24. مطلق

**ثانيا: الامراض المزمنة**

1. **هل تعاني من أي من الامراض المزمنة التالية؟**

|  | **المرض** | **نعم** | **لا** |
| --- | --- | --- | --- |
|  | **السكري** |  |  |
|  | **ارتفاع ضغط الدم** |  |  |
|  | **امراض قلب** |  |  |
|  | **امراض رئوية** |  |  |
|  | **التهاب المفاصل او الروماتيزم** |  |  |
|  | **سرطان** |  |  |
|  | **امراض تنفسية** |  |  |
|  | **امراض مزمنة اخري اذكرها:** | | |

**ثالثا:الوضع الصحي العام**

1. بشكل عام كيف تقيم صحتك؟ اختار إجابة واحدة فقط
2. ممتازة
3. جيدة جدا
4. جيدة
5. مقبولة
6. ضعيفة

**رابعا:اعراض مرضية**

**خلال الأسبوعين الماضيين**

|  | العرض | ابدا | قليل جدا | بعض الوقت | قليل من الوقت | معظم الوقت | دائما |
| --- | --- | --- | --- | --- | --- | --- | --- |
| 8 | هل عانيت من اية مشكلة صحية اثرت عليك؟ |  |  |  |  |  |  |
| 9 | هل كنت خائفا على وضعك الصحي في المستقبل؟ |  |  |  |  |  |  |
| 10 | هل انت قلقا على وضعك الصحي؟ |  |  |  |  |  |  |
| 11 | هل انت محبط من وضعك الصحي؟ |  |  |  |  |  |  |

*12.خلال الأسبوعين الماضيين هل عانيت من ضيق في النفس؟ يرجى اختيار رقم مناسب لإجابتك*

**|______|______|______|______|______|______|______|______|______|______|**

**0 1 2 3 4** 5 **6 7 8 9** 10

0**=لا يوجد ضيق نفس 5= ضيق نفس متوسط 10= ضيق نفس شديد**

*13.خلال الأسبوعين الماضيين هل عانيت من الم؟ يرجى اختيار رقم مناسب لإجابتك*

**|______|______|______|______|______|______|______|______|______|**

**0 1 2 3 4** 5 **6 7 8 9** 10

**0=لا يوجد الم 5= الم متوسط 10= الم شديد**

**خامسا:النشاط البدني**

**14. خلال الأسبوع الماضي كم من الوقت استغرقت لعمل الأنشطة البدنية التالية؟**

|  |  | **ابدا** | **اقل من 30 دقيقة** | **30-60 دقيقة** | **1-3 ساعات** | **أكثر من 3 ساعات** |
| --- | --- | --- | --- | --- | --- | --- |
|  | **تمارين شد ومد ورفع الجسم** |  |  |  |  |  |
|  | **رياضة المشي** |  |  |  |  |  |
|  | **سباحة والعاب مائية** |  |  |  |  |  |
|  | **البايسكل او أجهزة الرياضة** |  |  |  |  |  |
|  | **رياضة الايروبكس (حرق الدهون)** |  |  |  |  |  |
|  | **رياضات اخري اذكرها** |  |  |  |  |  |

**سادسا:الثقة بالنفس حول فعل الأشياء**

**الرجا وضع دائرة حول الرقم الذي يدل على مدى ثقتك بنفسك عند عمل المهام الاعتيادية التالية**

15.عدم جعل التعب الناجم عن مشكلة صحية من التاثير على أي عمل تنوي عملة

**|______|______|______|______|______|______|______|______|______|______|**

**0 1 2 3 4** 5 **6 7 8 9** 10

**عدم ثقة ثقة تامة**

16.عدم جعل الالم الناجم عن مشكلة صحية من التأثير على أي عمل تنوي عملة

**|______|______|______|______|______|______|______|______|______|______|**

**0 1 2 3 4** 5 **6 7 8 9** 10

**عدم ثقة ثقة تامة**

17.عدم جعل الاضطراب العاطفي الناجم عن مشكلة صحية من التاثير على أي عمل تنوي عملة

**|______|______|______|______|______|______|______|______|______|______|**

**0 1 2 3 4** 5 **6 7 8 9** 10

**عدم ثقة ثقة تامة**

18.عدم جعل اية اعراض مرضية ناجمة عن مشكلة صحية من التاثير على أي عمل تنوي عملة

**|______|______|______|______|______|______|______|______|______|______|**

**0 1 2 3 4** 5 **6 7 8 9** 10

**عدم ثقة ثقة تامة**

19.القيام بجميع الأنشطة والمهام لتحسين وتمكين وضعك الصحي التي تبعدك عن الذهاب للطبيب

**|______|______|______|______|______|______|______|______|______|______|**

**0 1 2 3 4** 5 **6 7 8 9** 10

**عدم ثقة ثقة تامة**

20.القيام بأعمال أخرى غير تناول الادوية لتخفيف من اثر المرض على حياتك اليومية

**|______|______|______|______|______|______|______|______|______|______|**

**0 1 2 3 4** 5 **6 7 8 9** 10

**عدم ثقة ثقة تامة**

**سابعا: الأنشطة اليومية خلال الأسبوعين الماضيين**

|  |  | **ابدا** | **قليلا** | **متوسطا** | **عاليا** | **عاليا جدا** |
| --- | --- | --- | --- | --- | --- | --- |
| **21** | **هل وضعك الصحي اثر على علاقاتك العائلية او الاجتماعية؟** |  |  |  |  |  |
| **22** | **هل وضعك الصحي أثر على هواياتك والأنشطة الترفيهية؟** |  |  |  |  |  |
| **23** | **هل وضعك الصحي اثر على واجباتك المنزلية؟** |  |  |  |  |  |
| **24** | **هل وضعك الصحي آثر على ذهابك للتسوق؟** |  |  |  |  |  |

**ثامنا:الرعاية الطبية**

**عندما تقوم بزيارة الطبيب كم مرة تفعل ما يلي (الرجاء اختيار إجابة واحدة)**

|  |  | **0**  **ابدا** | **1**  **على الاغلب لا** | **2**  **احيانا** | **3**  **في كثير من الاحيان** | **4**  **غالبا** | **4**  **دائما** |
| --- | --- | --- | --- | --- | --- | --- | --- |
| **25** | **تحضير مجموعة من الأسئلة للطبيب** |  |  |  |  |  |  |
| **26** | **تسأل عن أشياء تريد ان تعرفها لتفهم اكثر عن العلاج** |  |  |  |  |  |  |
| **27** | **مناقشة اية مسألة شخصية تؤثر على وضعك الصحي** |  |  |  |  |  |  |

**28. كم مرة قمت بزيارة الطبيب خلال ال 6 اشهر الماضية ما عدا الزيارات الطارئة؟**

**29. كم مرة قمت بزيارة الطبيب خلال ال 6 اشهر الماضية زيارات طارئة؟**

**30. كم مرة أدخلت الى المستشفى خلال ال6 اشهر الماضية لليلة واحدة او اكثر؟**

**انتهت الاستبانة شكرا لحسن تعاونكم**
