# Supplementary material for: Prevalence and associated factors of non-communicable chronic diseases among university academics in Jordan
Source: PLoS One. 2024 Aug 13;19(8):e0304829. doi: 10.1371/journal.pone.0304829 (PMC11321547; doi:10.1371/journal.pone.0304829)
Supplement: S3 File — (DOC) [file pone.0304829.s003.doc]

**A Questionnaire About Assessing Prevalence and associated factors of Chronic Diseases among University Academics in Jordan**

**Assessing your health status, particularly when dealing with chronic illnesses, can be challenging. We aim to understand your health status, whether you have one or multiple chronic diseases, or if you're generally healthy. Your response will be kept confidential, and we won't share it with anyone.**

**First: Demographic Characteristics**

**1. Gender:**

a). Male

b). Female

**2. Age:**

a) 20-30 years

b) 31-40 years’ old

c) 41-60 years

d) 60+ years old.

**3. Weight: Height:**

**4. Scientific rank:**

a) Professor Doctor

b) Associate Professor

c) Assistant Professor

d) Teacher

e) Research and teaching assistant

f) Others, mention them:

**5. Academic degree:**

a) Bachelor’s degree

b) Master's degree

c) Ph.D.’s. degree

**6. Marital status:**

1. Married
2. Single
3. Divorce
4. Widowed

**Second: Chronic Diseases**

**If you suffer from one or more of the following chronic diseases in the table, please specify it**

| **No.** | **Disease** | **Yes** | **No** |
| --- | --- | --- | --- |
|  | Diabetes Mellitus |  |  |
|  | Hypertension |  |  |
|  | Heart disease |  |  |
|  | Lung diseases |  |  |
|  | Rheumatoid Arthritis |  |  |
|  | Cancer |  |  |
|  | Respiratory diseases |  |  |
| **Other chronic diseases, mention them:** | | | |

**Third: General Health**

**In general, would you say your health is: (Circle one)**

Excellent……………. 1

Very good……………. 2

Good …………. 3

Fair…………... 4

Poor…………. 5

|  | | **Fourth: Symptoms** |  | | | | |
| --- | --- | --- | --- | --- | --- | --- | --- |
|  | **How much time during the past 2 weeks...** |  |  |  |  |  |  |
|  | **None** | **A little** | **Some** | **A good** | **Most** | **All** |  |
|  | **of the** | **of the** | **of the** | **bit of the** | **of the** | **of the** |  |
|  | **time** | **time** | **time** | **time** | **time** | **time** |  |
|  | 1. Were you discouraged by your  health problems? 0 | 1 | 2 | 3 | 4 | 5 |  |
|  | 2. Were you fearful about your  future health? 0 | 1 | 2 | 3 | 4 | 5 |  |
|  |  |  |  |  |  |  |  |
|  | 3. Was your health a worry in your life? 0 | 1 | 2 | 3 | 4 | 5 |  |
|  | 4. Were you frustrated by your  health problems? 0 | 1 | 2 | 3 | 4 | 5 |  |

1. We are interested in learning whether or not you are affected by fatigue. Please *circle* the *number* below that describes your **fatigue** in the **past 2 weeks:**

**
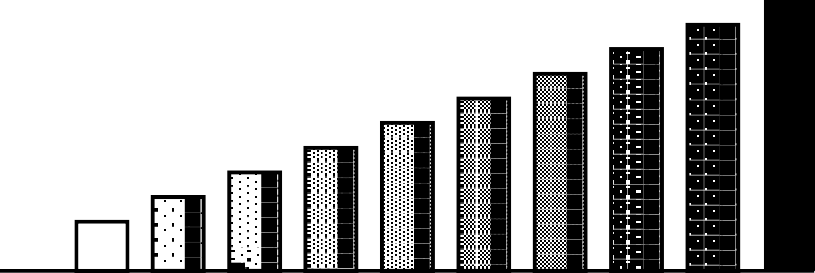
**

0 1 2 3 4 5 6 7 8 9 10

No Severe

fatigue fatigue

1. We are interested in learning whether or not you are affected by shortness of breath. Please *circle* the

*number* below that describes your **shortness of breath** in the **past 2 weeks:**

**
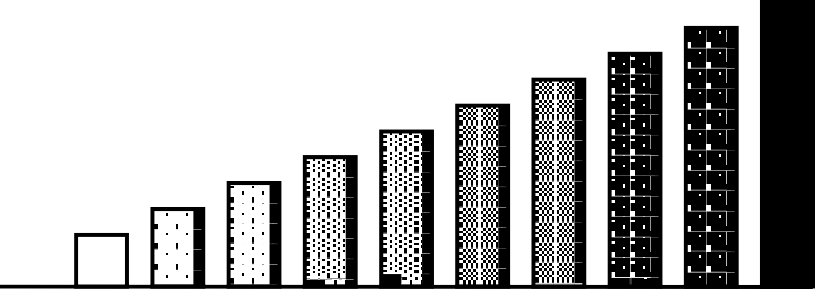
**

| 0 1 | 2 | 3 | 4 | 5 | 6 | 7 | 8 | 9 10 |
| --- | --- | --- | --- | --- | --- | --- | --- | --- |
| No  shortness |  |  |  |  |  |  |  | Severe  shortness |
| of breath |  |  |  |  |  |  |  | of breath |

1. We are interested in learning whether or not you are affected by pain. Please *circle* the *number* below that describes your **pain** in the **past 2 weeks.**

**
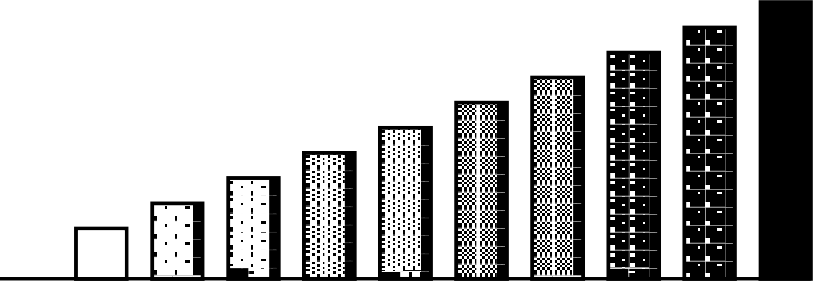
**

0 1 2 3 4 5 6 7 8 9 10

No Severe

pain pain

**Fifth: Physical Activities**

**During the past week,** even if it was not a typical week for you, how much **total** time *(for the* ***entire week****)* did you spend on each of the following? *(Please circle* ***one*** *number for each question.)*

| **None**  1. Stretching or strengthening exercises | **less than 30 min/wk** | **30-60**  **min/wk** | **1-3 hrs per week** | **more than 3 hrs/wk** |
| --- | --- | --- | --- | --- |
| (range of motion, using weights, etc.) 0 | 1 | 2 | 3 | 4 |
| 2. Walk for exercise 0 | 1 | 2 | 3 | 4 |
| 3. Swimming or aquatic exercise 0 | 1 | 2 | 3 | 4 |
| 4. Bicycling (including stationary  exercise bikes) 0 | 1 | 2 | 3 | 4 |
| 5. Other aerobic exercise equipment  (Stairmaster, rowing, skiing machine, etc.) 0 | 1 | 2 | 3 | 4 |
| 6. Other aerobic exercise *Specify* | 1 | 2 | 3 | 4 |

**Sixth: Confidence about Doing Things**

**For each of the following questions, please *circle* the number that corresponds with your confidence that you can do the tasks regularly at the present time.**

# How confident are you that you can...?

# Keep the fatigue caused by your disease from interfering with the things you want to do?

| 1 | 2 | 3 | 4 | 5 | 6 | 7 | 8 | 9 | 10 |
| --- | --- | --- | --- | --- | --- | --- | --- | --- | --- |

# 1=Not at all confident 10= Totally confident

# Keep the physical discomfort or pain of your disease from interfering with the things you want to do?

| 1 | 2 | 3 | 4 | 5 | 6 | 7 | 8 | 9 | 10 |
| --- | --- | --- | --- | --- | --- | --- | --- | --- | --- |

# 1=Not at all confident 10= Totally confident

# Keep the emotional distress caused by your disease from interfering with the things you want to do?

| 1 | 2 | 3 | 4 | 5 | 6 | 7 | 8 | 9 | 10 |
| --- | --- | --- | --- | --- | --- | --- | --- | --- | --- |

# 1=Not at all confident 10= Totally confident

# Keep any other symptoms or health problems you have from interfering with the things you want to do?

| 1 | 2 | 3 | 4 | 5 | 6 | 7 | 8 | 9 | 10 |
| --- | --- | --- | --- | --- | --- | --- | --- | --- | --- |

# 1=Not at all confident 10= Totally confident

# Do the different tasks and activities needed to manage your health condition so as to reduce your need to see a doctor?

| 1 | 2 | 3 | 4 | 5 | 6 | 7 | 8 | 9 | 10 |
| --- | --- | --- | --- | --- | --- | --- | --- | --- | --- |

# 1=Not at all confident 10= Totally confident

# Do things other than just taking medication to reduce how much your illness affects your everyday life?

| 1 | 2 | 3 | 4 | 5 | 6 | 7 | 8 | 9 | 10 |
| --- | --- | --- | --- | --- | --- | --- | --- | --- | --- |

# 1=Not at all confident 10= Totally confident

|  | **Seventh: Daily Activities** |  | | | |
| --- | --- | --- | --- | --- | --- |
|  | During the **past 2 weeks**, how much... *(Circle* ***one****)*   |  |  | Not at all | Slightly | Moderately | Quite a bit | Almost totally | | --- | --- | --- | --- | --- | --- | --- | | 1 | Has your health interfered with your normal social activities with family, friends, neighbors or groups? |  |  |  |  |  | | 2 | Has your health interfered with  your hobbies or recreational activities? |  |  |  |  |  | | 3 | Has your health interfered  with your household chores? |  |  |  |  |  | | 4 | Has your health interfered with  your errands and shopping? |  |  |  |  |  | |  |  |  |  |

**Eighth: Medical Care**

1. When you **visit your doctor**, how often do you do the following *(please circle* ***one*** *number for each question):*

| **Never**  a. Prepare a list of questions | **Almost never** | **Some- times** | **Fairly often** | **Very often** | **Always** |
| --- | --- | --- | --- | --- | --- |
| for your doctor 0 | 1 | 2 | 3 | 4 | 5 |
|  |  |  |  |  |  |
| b. Ask questions about the things you want to know and things you don’t  understand about your treatment 0 | 1 | 2 | 3 | 4 | 5 |
|  |  |  |  |  |  |
| c. Discuss any personal problems that  may be related to your illness 0 | 1 | 2 | 3 | 4 | 5 |

1. **In the past 6 months**, how many times did you visit a physician?

*Do* ***not*** *include visits while in the hospital or the hospital emergency department*... visits

1. **In the past 6 months**, how many times did you go to

a **hospital** emergency department? times

1. **In the past 6 months**, how many TIMES were you hospitalized

for one night or longer? times

- 1. How many total NIGHTS did you spend in the hospital **in the**

**past 6 months**? nights

- 1. Were any of these hospitalizations at a skilled nursing facility,

convalescent hospital, or other minimum care facility? *(circle)* ......................... Yes No

***Thank you for your help!***
