# Supplementary material for: Prevalence and associated factors of non-communicable chronic diseases among university academics in Jordan
Source: PLoS One. 2024 Aug 13;19(8):e0304829. doi: 10.1371/journal.pone.0304829 (PMC11321547; doi:10.1371/journal.pone.0304829)
Supplement: Attachment — Submitted filename: Response to Reviewers.doc [file pone.0304829.s004.doc]

**Response to Reviewers**

| **Reviewer 1 Comments** | | |
| --- | --- | --- |
| **Comments** | | **Author Response** |
| Suggestion 1 | I suggest to edit the title as follow: Prevalence and Associated Factors of Chronic Diseases Among University Academics in Jordan. | Accepted and changed |
| Suggestion 2 | Line 146: The term "subjects" may not be appropriate in this context, as it can imply a level of passivity. Consider using "Participants" or "Study Participants" for clarity and to maintain professionalism. | Accepted and changed |
| Suggestion 3 | Line 162: Provide more detail about the self-administered structured questionnaire, such as the number of questions, response options, and any specific chronic diseases addressed. This will enhance the understanding of the instrument's content and relevance to the study. | Accepted and More details added |
| Suggestion 4 | Line 174: Specify the qualifications and expertise of the professional translator who translated the questionnaire into Arabic to ensure accuracy and reliability in the translation process. | Accepted and Specified |
| Suggestion 5 | Line 202: Specify whether the written consent form provided to participants was available in both Arabic and English to accommodate diverse language preferences among the academic staff. | Written consent was Specified |
| Comment 1 | Although the study mentions the participation of a majority of faculty members, it is crucial to include specifics about the sample size and the proportion of faculty members who were approached but declined to participate. Furthermore, the study should address whether the sample accurately represents the broader academic population at Mutah University. | Because Mutah University's academic staff is relatively small, comprising 612 academics, all academic members were surveyed for the study. As a result, there was no need to select a sample from this population. Surveying the accessible population ensures accuracy and precision without any margin of error, aside from potential inaccuracies in respondents' answers. |
| Comment 2 | The specified timeline for data collection (February 5 to March 5, 2023) provides clarity regarding the study's duration. However, it would be beneficial to discuss any considerations regarding seasonal variations or academic schedules that could impact participant availability or response rates during this period. | This timeframe was specifically selected because it signaled the start of the second semester, when all academic personnel were fully engaged in their duties following the approval of the teaching materials program by the Admissions and Registration Unit. Additionally, the teaching divisions had stabilized after the withdrawal and addition period. |
| Comment 3 | The discussion underscores significant gender disparities in smoking prevalence, noting a higher percentage of male participants reporting smoking compared to females. However, it would be beneficial to explore the underlying reasons for these disparities further, taking into account factors such as cultural norms, social attitudes towards smoking, and gender-specific health behaviors. | In Jordanian society, smoking is more commonly accepted among men than women, often due to societal ideals of masculinity and femininity. Smoking might signify status or defiance for men, whereas women often encounter greater social disapproval. Furthermore, variances in health behaviors between genders are influenced by upbringing, healthcare accessibility, and individual perceptions of health risks. For instance, coping mechanisms for stress and willingness to seek assistance for smoking cessation may differ between men and women. Additionally, designing health campaigns specifically targeted at each gender could yield differing effects on smoking behaviors. |
| **Reviewer 2 Comments** | | |
| Comment 1 | Lines 50-53: The two sentences in lines 50-53 might be redundant. Please review them. | Reviewed and modified (Chronic illnesses such as diabetes, cancer, and cardiovascular conditions are the primary contributors to mortality in the United States, accounting for over two-thirds of all deaths.) |
| Comment 2 | Lines 64-67: This part includes a long confusing sentence that needs to be rewritten. | Rewritten (The prevalence of diabetes among individuals aged 25 and above in Jordan is notably high, affecting 34%. Forecasts indicate a significant increase in the overall prevalence of chronic diseases in the country from 2005 to 2050, mirroring this concerning trend.) |
| Comment 3 | Lines 76-77: A reference is needed for the following sentence “Despite the widespread belief that university academics generally maintain good health”. | Reference (17) inserted |
| Comment 4 | Lines 96-98: the sentence is lengthy, unclear, and includes a new abbreviation NCDs! | Rewritten (In the Eastern Mediterranean Region (EMR), which encompasses Jordan, Non-Communicable Diseases (NCDs) pose a significant burden, with mortality rates exceeding those of communicable diseases by more than 10 times.) |
| Comment 5 | Lines 106-110: Thesis-like summary at the end of the introduction is undesirable. | Deleted |
| Comment 6 | Lines 111-117: Subtitles under the research questions are not needed. Be more concise and just mention the research questions. | Modified |
| Comment 7 | Lines 118-122: Once again, a thesis-like summary is not appropriate in research articles. | Deleted |
| Comment 8 | Line 131: Do we need to know the area of the university, does it matter? | Deleted |
| Comment 9 | Lines 132-137: Instead of mentioning the faculties by name, it is worth noting that classifying faculties into health and non-health faculties might bring up some significant and more meaningful differences. | Modified |
| Comment 10 | Lines 138-145: This paragraph introduces extra information about students, better to remove. | Removed |
| Comment 11 | Although the researchers studied academics in Jordan who are apparently older than 40 years old, the introduction introduces the prevalence of chronic diseases among young people. The introduction needs to be enriched with more information and statistics about NCCDs among older people. | One paragraph regarding chronic diseases among elderly added (Across the globe, the elderly population is disproportionately affected by chronic illnesses. Statistics reveal that between 80% to 92% of older adults have at least one chronic disease, while 50% to 77% have two or more. The prevalent chronic conditions among the elderly include cardiovascular disease, chronic obstructive pulmonary disease, and diabetes.) |
| Comment 12 | Throughout the manuscript, the researchers keep mentioning chronic diseases despite introducing the NCCDs term at the beginning of the introduction. Can you use the term NCCDs instead of just repeating the names of chronic diseases? | Chronic diseases term changed to NCCDs throughout the manuscript |
| Comment 13 | Did you use a priori or post hoc analysis to estimate the sample size? Doing so is necessary to draw valid conclusions. | Sample size estimation wasn't conducted through either a priori or post hoc analysis since the entirety of the target population was recruited, and no sampling technique was employed. |
| Comment 14 | Line 240: Suddenly, a new term (Pain) was introduced! This is very far from the study's aims. | "Pain is listed within the section dedicated to symptoms of chronic diseases in the Sample Questionnaire for Chronic Disease, which was created by the Stanford Patient Education Research Center." The following sentence added( Concerning the primary symptoms associated with NCCDs, pain was observed among the participants who were asked to gauge the intensity of their pain during the previous two weeks using numerical pain rating scales (NPRS).) |
| Comment 15 | Table 1: it was mentioned in the methodology that both Jordanian and Non-Jordanian academics were included in the study. However, table 1 doesn't show this classification. | Even though the count of non-Jordanian faculty members at the university is minimal, akin to the number of fingers on one hand, the questionnaire was still distributed to them. However, upon analyzing the data, it became evident that all participants were Jordanian. |
| Comment 16 | Subheadings under the results are preceded by unnecessary words (part1, part2, and part 3). | Modified |
| Comment 17 | Did you follow the STROBE checklist in reporting your research? Please add it as a supplementary file. | Yes ,added and uploaded |
| Comment 18 | The quality of figure one is poor, a new figure with high resolution is needed to be produced | Changed |
| Comment 19 | Reference list: Mistakenly, a full stop appears before the number of each reference. | Full stop deleted |
| **Reviewer 3 Comments** | |  |
| Comment1 | The discussion identifies a high prevalence of regular walking among academic staff, attributing it to awareness of the importance of physical activity in preventing chronic diseases. While this interpretation is plausible, further discussion on potential barriers to physical activity among academic staff, such as time constraints or work-related stress, would provide a more nuanced understanding of the findings. | Added (Despite the busy schedules of academic staff, encompassing teaching, research, administrative tasks, and other duties, our study found that 81.6% of academics engage in regular walking. This rate exceeds the 12.5% reported in a national study in Jordan, reflecting a higher level of physical activity among academics. This increased activity could be due to their understanding of its role in preventing chronic diseases. Furthermore, the availability of suitable facilities and walking paths on campus may contribute to this elevated level of physical activity among academics. |
| Comment2 | The discussion briefly mentions the importance of maintaining a healthy body weight and engaging in regular physical activity to prevent chronic diseases. However, offering specific recommendations for future research directions and interventions aimed at promoting health and reducing the prevalence of chronic diseases among academic staff would enhance the discussion's practical implications. | Added (Specific suggestions for future research and interventions aimed at promoting health and reducing chronic disease rates among academic staff could include conducting longitudinal studies to evaluate the impact of workplace wellness programs tailored to this population. Additionally, implementing educational workshops focusing on nutrition and stress management, along with incorporating regular physical activity breaks into the daily work schedule, presents promising strategies for exploration and implementation. These targeted initiatives not only have the potential to enhance the health and well-being of academic staff but also to cultivate a supportive and health-conscious work environment. |
| Comment 3 | It is noted that specific items in the questionnaire are subjective and rely on each participant's individual opinions. While subjectivity in questionnaire items is common, it's essential for researchers to acknowledge this limitation and consider potential biases introduced by subjective responses. | Modified “Recognizing the constraints of subjective questionnaire items and the biases they might bring forth allows researchers to actively refine research methodologies. This understanding can motivate future investigations to seek alternative strategies, like mixed methods research or triangulating data sources, in order to mitigate subjectivity's impact and improve the validity of research outcomes.” |
| Comment 4 | The implications could explore how the study findings could be integrated into the nursing education curriculum to prepare future nurses for addressing chronic diseases in community settings. | **Added to the implications** “Incorporating research findings into nursing education curriculum is essential for equipping future nurses to adeptly manage NCCDs within communities. This may entail arranging health fairs, facilitating educational workshops, engaging in community-based research initiatives, and advocating for policies that enhance health equity and improve access to care for individuals grappling with NCCDs.” |
