# Supplementary material for: Prevalence and associated factors of non-communicable chronic diseases among university academics in Jordan
Source: PLoS One. 2024 Aug 13;19(8):e0304829. doi: 10.1371/journal.pone.0304829 (PMC11321547; doi:10.1371/journal.pone.0304829)
Supplement: Attachment — Submitted filename: Response to reviewrs 2-5-2024.doc [file pone.0304829.s005.doc]

| **Response to Editor and Reviewer 2 Comments:**  **I express my sincere gratitude to the editor for his insightful comments. I'm hoping my responses to them will be acceptable and persuasive.** | | |
| --- | --- | --- |
| **Comments** | | **Author Response** |
| Comment 1 | Many paragraphs of the manuscript seem like AI-Assisted Language/writing, please confirm if AI software was used or not. | Initially, the manuscript underwent review by an English language editor. However, in response to reviewers comments, a software application was utilized to validate the modifications made, specifically for proofreading purposes rather than generating the text. |
| Comment 2 | The manuscript needs extensive English language editing. | The English language editing was carried out by a professor who specializes in the English language. |
| Comment 3 | Line 95-100: Rephrase the sentence without using first person pronouns | The requested paragraph (line 95-100) does not apply to the said comment, but the only relevant paragraph is the paragraph between lines 82-87 and it has been amended as follow:” Being a researcher training nursing students in hospitals and medical centers exposed a different reality. Academic colleagues regularly receiving monthly medications for various NCCDs were consistently observed. As an associate professor employed in a health faculty, this observation motivates to initiate a study on the health status of academics in similar environments. Consequently, the purpose of this study is to assess the NCCDs and related factors among academic staff at Mutah University in Jordan. The findings aim to offer valuable insights for intervention planning and stakeholder engagement. |
| Comment 4 | Lines 104 – 126: I do not understand the rationale behind these sections as they both contains repetitive information which was already provided in the introduction | Thank you for this comment. You mean lines 104-106. The two sentences deleted and another paragraph added |
| Comment 5 | Study instruments: The authors explain that they adopted the Sample Questionnaire for Chronic Disease from Stanford Patient Education Research Center. Did the author use part or all the questionnaire items? The authors should clarify this fact and they have to explain to the readers the operationalization of the only items used or adopted from the questionnaire to avoid confusion. How did the authors measure BMI to identify obesity vs overweight? Did the survey include body weight and height? Also, why alcohol use was not examined as it is a potential risk factor? Also, how did the author define regular walking? Any criteria? | - All the original questionnaire items from Stanford Patient Education Research Center used in the data collection. However, as the focus of this manuscript was solely on questions that were directly relevant to the research title, objectives, and questions, certain items were overlooked by data analysis and reported in the finding’s section. - BMI was calculated based on weight and height reported by the participant which included in the SPSS data file. Apologize for sending an old Arabic version of the survey that it does not include it. Please check the new updated Arabic Version which already we used in the data collection. - Alcohol is not included in the questionnaire and not within the focus of this study. - Those who answered the question about walking and their answer was walking for more than 3 hours a week, meaning half an hour a day, is considered regular walking. This is added to the text. Thank you for noticing this point. |
| Comment 6 | Discussion Section: While comparing the findings with regional studies, are the comparison between countries involves studies among academics? Comparing your study findings which is a single-center study among academics with other regional studies that involve various study populations will not be reliable. Please modify the discussion to compare your findings with regional or international studies that examined NCCDs among academic staff. | There is no literature specifically on academics to compare the results of this study with. Therefore, the comparison applied with various populations reported in other studies. I understand this may impose bias in the interpretation. Furthermore, it is clarified in the discussion section and added as a limitation. |
| Comment 7 | Limitations: Line 423-424: Better to be omitted | Thank you for this comment. You mean lines 323-324. Deleted as suggested. |
| Comment 8 | Implications: Unfortunately, the implications provided do not match the overall objective of the study. Why would the authors direct the implications toward nurses’ education only? I know that this was respectfully suggested by one of the reviewers, however, discuss in this section the potential consequences, recommendations, or practical applications of research findings without specifying nurses’ roles per se. | I agree with you this should not directed toward nurses. However, the role of all health professionals cannot be ignored. Therefore, the implications amended to reflect education, research and practice of all health professionals. |
| Comment 9 | The authors are attaching the Stanford Survey only. Please provide the survey that the authors formulated to collect the data in this study for replicability and not the Stanford questionnaire (as it is already available publicly). | English and Arabic versions used in the study uploaded as requested. |
| **Reviewer 2 Comments** | | |
|  | Reviewer #2: Thank you for addressing most of the concerns and comments I raised. However, the article still lacks for the STROBE checklist with proper citation. | The proper citation added as following: Gallo V, Egger M, McCormack V, Farmer PB, Ioannidis JPA, et al. (2011) STrengthening the Reporting of OBservational studies in Epidemiology – Molecular Epidemiology (STROBE-ME): An Extension of the STROBE Statement. PLOS Medicine 8(10): e1001117. https://doi.org/10.1371/journal.pmed.1001117 |
